# Immunohistochemical Expression of BCL-2 in Endometrial Carcinoma and Its Comparison With Hormone Receptor Status and Epidermal Growth Factor

**DOI:** 10.7759/cureus.33346

**Published:** 2023-01-04

**Authors:** Sharanya Kandaswamy, Poongothai Palanisamy

**Affiliations:** 1 Department of Pathology, Kovai Medical Center and Hospital Institute of Health Sciences and Research, Coimbatore, IND; 2 Pathology, Eureka Pathology Laboratory, Coimbatore, IND

**Keywords:** progesterone receptor, cancer, hormone receptors, estrogen receptor, endometrial carcinoma

## Abstract

Background: Endometrial carcinoma is the most common malignant tumor of the female genital tract with increasing incidence in developed countries. In the era of targeted therapy, immunohistochemical markers play an important role in the treatment and prognosis of endometrial carcinoma. The aim of the study was to study the immunohistochemical expression of B-cell lymphoma 2 (BCL-2) in endometrial carcinoma and to study the correlation of BCL-2 expression with hormone receptor status and transforming growth factor receptors in endometrial carcinoma.

Methods: Endometrial carcinoma reported between the period from January 2010 to December 2014 in the department of pathology of this institute was considered in the study. The study included cases of endometrial carcinoma reported on both curetting and hysterectomy specimens. In the samples where both curetting and hysterectomy were received only hysterectomy blocks were included and the curetting was excluded.

Results: The total number of malignancies reported during this period was 3478. Of these 3478 malignancies, 59 were endometrial carcinomas with an incidence of 1.6%. Out of 59 endometrial carcinomas, 46 cases were diagnosed on hysterectomy specimens and 13 cases were diagnosed on endometrial curetting biopsies.

Conclusion: The positive expressions of estrogen receptor (ER) and progesterone receptor (PR) in endometrial carcinoma suggest that steroid receptor studies may be of potential benefit in the management of some patients with endometrial carcinoma.

## Introduction

Endometrial carcinoma is the most common malignancy of the female genital tract. It ranks fourth among malignancies occurring in women worldwide [1] and is the third most common cancer in South-Eastern Asia [2]. It occurs usually in post-menopausal women with a median age of 65 years, unless with genetic predisposition or high body mass index (BMI >30-40) [3].

Endometrial carcinoma is classified as type I and type II endometrial carcinoma. Type I endometrioid tumors are the most common tumors and 80% of the tumor belongs to this type. It occurs typically at 65 years of age or less and is low grade, present at an early stage with a good prognosis. These tumors usually are hormone-dependent. The occurrence of type II tumors is rare compared to type I tumors. They are high grade, aggressive in nature with poorer prognoses [4].

In the era of targeted therapy, immunohistochemical markers play an important role in the treatment and prognosis of endometrial carcinoma. Estrogen and progesterone receptors are important prognostic markers. The presence of these receptors in endometrial carcinoma is associated with a good prognosis.

Human epidermal growth factor receptor 2 (ERBB2, also known as HER2/neu) is a member of the epidermal growth factor receptor. HER2-neu mutation plays an important role in the pathogenesis of numerous cancers including endometrial carcinoma. Because of the invention of targeted therapy against mutated HER2/neu receptors, the identification of HER2 overexpression is essential. HER2 neu expression is seen in higher grade and advanced stages with poorer survival [5].

Apoptosis plays an important role in the development of cancer. B-cell lymphoma 2 (BCL-2) is a proto-oncogene that inhibits apoptosis and its dysfunction leads to malignancy. BCL-2 expression is associated with many tumors including carcinoma breast, prostate lung, and endometrium. Endometrial carcinoma is related to good prognosis and may play a vital role in the progression of growth from stage I to stage IV [6].

Treatment and longevity of life depend on staging and prognostic factors like estrogen receptor (ER), progesterone receptor (PR), HER2/neu, and BCL-2. We propose to study the immunohistochemical expression of BCL-2 in endometrial carcinoma reported from our institute and its correlation with hormone receptor status and transforming growth factor. This article is based on the thesis defended on September 12, 2013, in the "Tamil Nadu Dr. M.G.R. Medical University."

## Materials and methods

This was a retrospective observational study of the period from January 2010 to December 2014 in the department of Pathology at PSG Institute of Medical Sciences and Research (PSGIMSR), Coimbatore, Tamilnadu, India. The study included cases of endometrial carcinoma reported on both curettage and hysterectomy specimens. The samples from patients with a history of hormone treatment, chemotherapy, or radiation therapy were excluded.

The clinical details like patients’ age, parity, menstrual status, clinical staging, and gross specimen details were taken from the pathology requisition forms and from the medical records department of this institute after obtaining permission from the concerned authorities and institutional human ethics committee clearance.

The hematoxylin and eosin slides of these cases were retrieved and analyzed for assessing histopathological type of the tumor and tumor grading. The histological type of endometrial carcinoma was classified by the World Health Organization, and the staging of the tumor was done with the revised International Federation of Gynecology and Obstetrics (FIGO) system. All included slides had a minimum of 60-70% well-preserved tumor area with less than 10% of the necrotic area for immunohistochemistry. This was done to prevent staining artifact while doing immunohistochemical studies. Four thin (4-5 micron) sections were taken from representative blocks to perform immunohistochemical studies on ER, PR, BCL-2, and HER2/neu.

Immunohistochemistry for ER (monoclonal ER alpha, EP1), PR (monoclonal PR, EP2), and BCL-2 (BCL-2, EP 36) and HER2-neu (monoclonal HER2/ ERB 2- EP 3) from PathnSitu, USA, done using a supersensitive horse radish peroxidase (HRP) enzyme detecting system with 3,3-diaminobenzidine tetrahydrochloride (DAB) as chromogen. Antigen retrieval was done using ethylenediamine tetraacetic acid (EDTA) buffer at an alkaline pH of 9.0 and phosphate-buffered saline (PBS) was prepared with a pH value of 7.6 for washing. The stained slides were examined for the expression of BCL-2, HER2/neu, ER, and PR, and compared with controls (carcinoma breast-ER, PR, HER2/neu; lymph node: BCL-2).

Parameters like age and parity of the patient, menstrual status, clinical stage and tumor grade, histological type of tumor, myometrial invasion, and staining properties of ER, PR, BCL-2, and HER2/neu were entered into a Microsoft Excel worksheet, and a descriptive statistical analysis was performed on the same. Menopause was defined as a period of 12 months without prior menstrual period. Also, BCL-2 expression was correlated with ER, PR, and HER2/neu staining properties.

## Results

**Figure 1:**
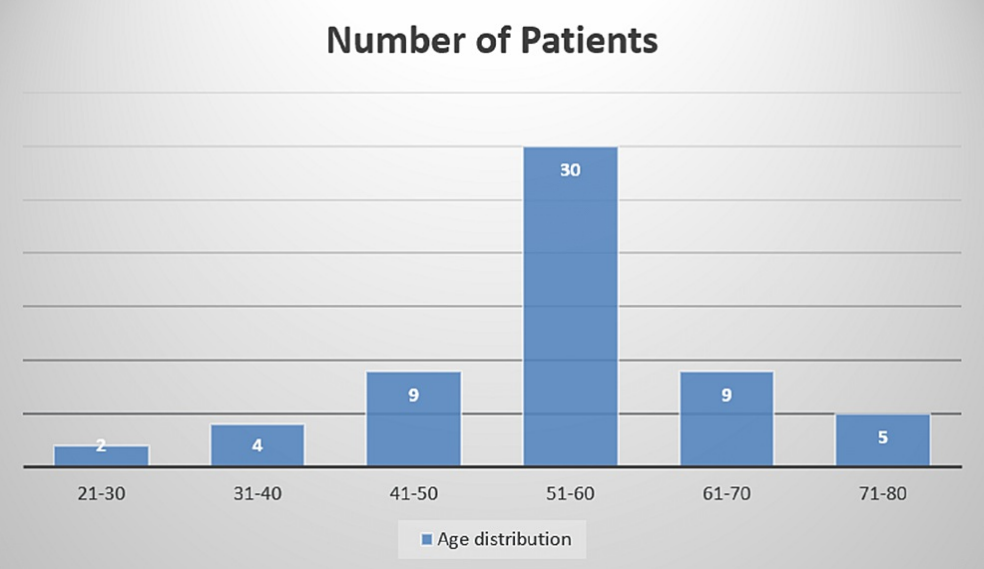
Age-wise distribution of the cases. Age is presented in years.

The Department of Pathology, PSG Institute of Medical Science and Research received 28626 biopsy specimens over a period of five years from January 2010 to December 2014. The total number of malignancies reported during this period was 3478. Of these 3478 malignancies, 59 were endometrial carcinomas with an incidence of 1.6%. Out of 59 endometrial carcinomas, 46 cases were diagnosed on hysterectomy specimens and 13 cases were diagnosed on endometrial curetting biopsies. These 13 cases were not subjected to hysterectomy and they were lost to follow-up. Out of 46 hysterectomy cases, 13 cases were diagnosed by endometrial curetting before hysterectomy, and those curetting biopsies were excluded from our study. An analysis of the study population showed that the mean age at diagnosis was 55 years. More than half of the cases were distributed between the age group of 51 and 60 years (Figure 1).

Table 1 showed that 42 cases of endometrial carcinoma occurred in women with a parity of three or less. Only two cases were nulliparous in our study. In the present study, 13 (22.03%) patients were perimenopausal and 46 (77.97%) patients were diagnosed after menopause. The most common histological type of endometrial carcinoma in our study was endometrioid type 55 (93%). Only four (7%) cases of endometrial carcinoma were non-endometrioid carcinoma (Table 1). Of the 59 cases, 35 (59.3%) of the endometrial carcinoma were International Federation of Gynecology and Obstetrics (FIGO) grade I, making it the commonest grade at presentation for endometrial carcinoma in our study (Table 1). Only four cases were of grade III as shown in Table 1. FIGO staging was done only in patients who underwent a hysterectomy (Table 1). Among the 46 hysterectomy specimens included in our study 33 (71%) cases were found to be in stage I as shown in Table 1.

**Table 1:** Clinical characteristics of the patients as well as their classification and staging of carcinoma of the endometrium. Data are presented as numbers (percentages). FIGO: International Federation of Gynecology and Obstetrics

Variables		Patients (%)
Parity	Parity of 3 and below	42 (71.18)
	Parity of 4 and above	15 (25.42)
	Nulliparous	2 (3.38)
Menstrual status	Postmenopausal women	46 (77.97)
	Perimenopausal women	13 (22.03)
Type of endometrial carcinoma	Endometrioid carcinoma	55 (93)
	Non-endometrioid carcinoma	4 (7)
Histologic grade	Grade I	35 (59.3)
	Grade II	20 (33.8)
	Grade III	4 (6.7)
FIGO stage at diagnosis	Stage I	33 (71)
	Stage II	6 (13)
	Stage III	5 (10)
	Stage IV	2 (4.3)

**Figure 2:**
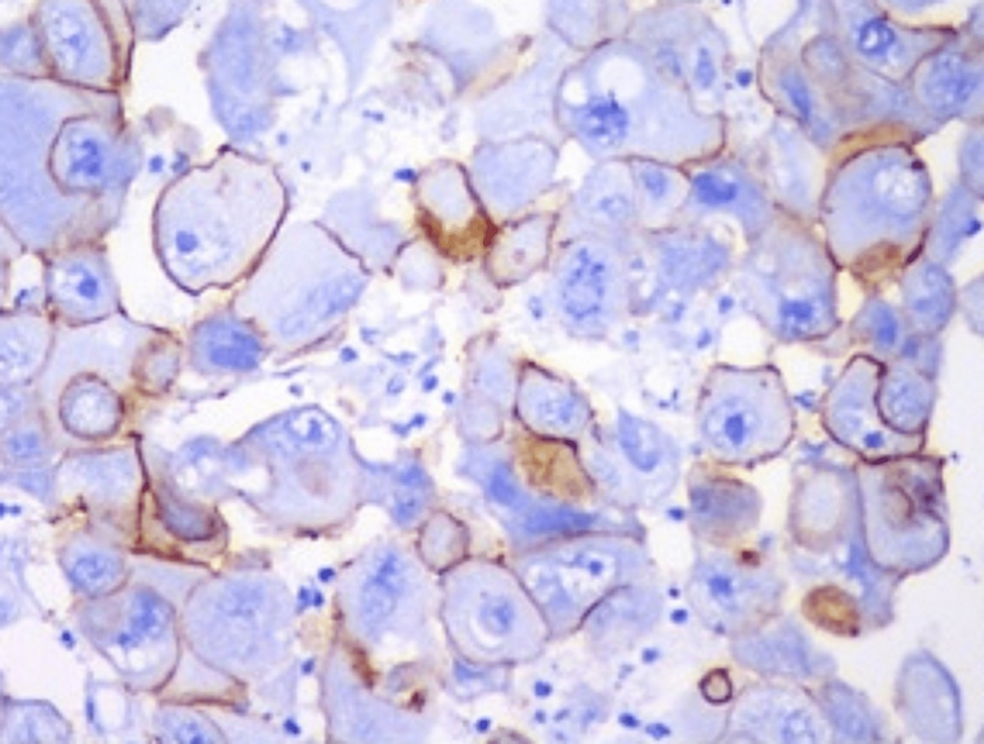
HER2/neu positivity (IHC, 400x). HER2/neu: human epidermal growth factor receptor 2; IHC: immunohistochemistry

Table 2 depicts that among the 59 cases of endometrial carcinoma, 33 (55.93%), 44 (74.57%), six (10.10%), and 38 (64.40%) patients were observed to be ER, PR, HER2/neu, and BCL-2 receptor positive (Figure 2). However, the maximum number of receptor-negative patients was observed to be for HER2/neu (Table 2).

**Table 2:** Number of endometrial carcinoma cases that are ER, PR, HER2/neu, and BCL-2 positive or negative. Data are presented as numbers (percentages). ER: estrogen receptor; PR: progesterone receptor; HER2/neu: human epidermal growth factor receptor 2; BCL-2: B-cell lymphoma 2

Expressions	Receptors			
	ER, n (%)	PR, n (%)	HER2/neu, n (%)	BCL-2, n (%)
Positive	33 (55.93)	44 (74.57)	6 (10.10)	38 (64.40)
Negative	26 (44.06)	15 (25.42)	53 (89.80)	21 (35.59)

Table 3 shows the number of cases with positive expressions of ER, PR, BCL-2, and HER2/neu. The maximum positive expression in endometroid adenocarcinoma was noted in progesterone receptor. All four (100%) non-endometrioid adenocarcinoma and two (3.63%) endometrioid adenocarcinoma revealed HER2/neu expressions (Table 3).

**Table 3:** Number of cases with types of endometrial carcinoma and expression of ER, PR, HER2/neu, and BCL-2. Data are presented as numbers (percentages). ER: estrogen receptor; PR: progesterone receptor; HER2/neu: human epidermal growth factor receptor 2; BCL-2: B-cell lymphoma 2

Type	ER positive, n (%)	PR positive, n (%)	HER2/neu positive, n (%)	BCL-2 positive, n (%)
Endometrioid adenocarcinoma (55 cases)	33 (60)	44 (80)	2 (3.63)	38 (69.09)
Non-endometrioid adenocarcinoma (four cases)	0	0	4 (100)	0

Table 4 shows that BCL-2 was more expressed in both ER, PR positive cases than in ER, PR negative cases. BCL-2 expression was directly related to ER, PR receptor positivity. HER2/neu was expressed in ER, PR negative cases only and it was not expressed in ER and PR positive cases (Table 4).

**Table 4:** Distribution of BCL-2, HER2/neu positivity in association with hormone receptor status. Data are presented as numbers (percentages). ER: estrogen receptor; PR: progesterone receptor; HER2/neu: human epidermal growth factor receptor 2; BCL-2: B-cell lymphoma 2

Expression	BCL-2 positive, n (%)	HER2/neu positive, n (%)
ER positive (33 cases)	25 (75)	0 (0)
ER negative (26 cases)	13 (50)	6 (23.07)
PR positive (44 cases)	34 (77.2)	0 (0)
PR negative (15 cases)	4 (26.6)	6 (40)

Table 5 reveals that 21, 28, and 25 of the cases that belong to FIGO stage I were positive for ER, PR, and BCL-2, respectively. Three cases were observed to be ER, PR, and BCL-2 receptor-positive in FIGO stage II (Table 5). Four (80%) and two (100%) cases were observed to be HER2/neu status positive in FIGO stage III and IV, respectively (Table 5).

**Table 5:** Number of cases ER, PR, HER2/neu, and BCL-2 expression with FIGO staging. Data are presented as numbers (percentages). ER: estrogen receptor; PR: progesterone receptor; HER2/neu: human epidermal growth factor receptor 2; BCL-2: B-cell lymphoma 2; FIGO: International Federation of Gynecology and Obstetrics

FIGO stage	ER positive	PR positive	HER2/neu positive	BCL-2 positive
Stage I (33 cases)	21 (63.63)	28 (84.8)	0 (0)	25 (75.75)
Stage II (6 cases)	3 (33.33)	3 (50)	0 (0)	3 (50)
Stage III (5 cases)	0 (0)	2 (40)	4 (80)	1 (20)
Stage IV (2 cases)	0 (0)	0 (0)	2 (100)	0 (0)

## Discussion

Malignancies constituted 12.1% of all the biopsies reported in our institute during the study period from January 2010 to December 2014. Endometrial carcinoma constituted 1.6% of all malignancies (59 over 3478) in this institute and is less than Chennai Cancer Registry Report (4.6%) in 2012 [7]. This is also much less than the global incidence of 14.7% as reported in Global Cancer Statistics in 2012 [1].

In India, endometrial carcinoma ranks third among all gynecological malignancies. The prognosis of the patient with endometrial carcinoma depends on the age at the time of diagnosis. The five-year survival rate is near 100% for carcinoma that occurs in women, particularly before menopause. Women in the premenopausal age group exhibit good histopathological features and are hormone-dependent. Survival reduces by 50% in women in the postmenopausal age group [8].

Endometrial carcinomas are classified as type I endometrioid adenocarcinoma and type II non-endometrioid adenocarcinoma. Type I endometrioid adenocarcinoma is common and occurs in the younger age group when compared to type II. Type I is usually associated with unopposed estrogen. Mostly they are low-grade and present at a lower stage with an excellent prognosis. These tumors are positive for estrogen and progesterone receptors. Type II non-endometrioid adenocarcinoma occurs in the older age group women and is not related to hormones. It is characterized by high grades and has a poorer prognosis [4].

In the era of targeted therapy, there is always a search for prognostic markers. As endometrial carcinomas are hormone-dependent, an immunohistochemical marker for estrogen, the progesterone receptor is commonly used prognostically. A few of the markers that are available for endometrial carcinoma include BCL-2 and HER2/neu. Estrogen and progesterone receptors are normally present in the endometrium. The presence of these receptors in endometrial carcinomas is associated with better prognosis and survival [9].

BCL-2 is a proto-oncogene that inhibits programmed cell death. In the endometrium, BCL-2 is very much expressed in the follicular phase with low expression during the secretory phase. BCL-2 expression sustains at high levels in typical hyperplasia. BCL-2 expression is progressively low in complex hyperplasia with atypia and with grade III, advanced endometrial carcinomas. BCL-2 expression is related to good prognosis and may play a vital role in tumor evolution from early to higher stages [6].

HER2/neu is one of a member of the epidermal growth factor receptor and plays an important role in the regulation of cell proliferation and differentiation. HER2/neu mutation has played a vital role in the treatment of endometrial carcinoma. Many studies support the association of HER2/neu overexpression with poorer outcomes in endometrial carcinoma [10].

The response to therapy is directly related to hormone receptor status, age, grade, stage at presentation, and also the availability of targeted therapy for mutation like HER2/neu receptors. With this background, we considered analyzing the hormone receptor status, BCL-2, HER2/neu mutation, and its correlation with various stages. We identified and retrieved paraffin blocks of 59 cases of endometrial carcinoma from the Archives of Pathology using inclusion and exclusion criteria as discussed earlier. Out of 59 cases, 46 cases are from hysterectomy specimens and 13 from curetting specimens.

Our study group comprising of women between the age group of 27 and 80 years. The majority of the cases occurred between the age group of 51 and 60 years with the mean age group of 55 years and this is similar to other studies [11]. More than 90% of cases belong to type I endometrioid adenocarcinomas. Type II non-endometrioid adenocarcinomas occurred in less than 10% of cases [12].

We graded both endometrioid and non-endometrioid tumors. For endometrioid tumors, grading was done using architectural features and nuclear changes. In our study group, 33 out of 59 cases (59.3%) were grade I. This is similar to the study conducted by Srijaipracharoen et al. which reported 57% showed grade I tumors [13].

Grade II tumors account for 33.8% which is similar to the data obtained from a study conducted by Sakuragi et al. [6]. All of the grade III tumors are non-endometrioid tumors and belong to the patient in the age group of 53-60 years. There is no positive correlation between the age group and the grade of tumors. FIGO staging was available only for hysterectomy patients. Most of the patients who underwent curetting were lost to follow-up after the diagnosis. Hence staging was not available for all cases of curetting included in the study.

Out of the 46 cases from hysterectomy specimens, 33 cases (71%) were found to be in stage I at the time of diagnosis, which is similar to the literature reported as 68.1% cases were present in stage I. Stage II tumors for 13%, stages III and IV account for 15.21% which was similar to the study reported in the literature as stage II were 18.1%, stages III and IV tumors were 13.6% [14].

Out of 59 cases taken in our study group, estrogen receptor was positive in 33 cases (55.9%) and negative in 26 cases (44.1%). The estrogen receptor positivity was low compared to the study conducted by Maniketh et al. which reported 73.3% of cases as estrogen receptor-positive [11]. We had 35 grade I tumors, 20 grade II tumors, and four grade III tumors. Twenty-five out of 35 cases of grade I tumors were positive for estrogen receptors which is similar to the literature that reported 72.2% of grade I tumors were estrogen positive [11]. Eight out of 20 grade II tumors (40%) were estrogen receptor-positive and all grade III tumors were estrogen negative.

In 33 cases with FIGO stage I, 21 cases (63.63%) which is similar to the study conducted by Srijaipracharoen et al. who reported that 62.4% of stage I-II tumors were estrogen positive [13]. Only 50% (three out of six) of stage II tumors were estrogen positive. All stage III and IV tumors were estrogen receptor-negative. In our study group, 44 out of 59 cases (74.57%) were progesterone receptor-positive. This value is 10% low compared to that reported in the literature, which showed 84.4% of PR positivity [11]. These receptors were positive in 33 out of 35 cases (94.28%) of grade I tumors and are higher than the study which showed 77.7% of grade I tumors were progesterone positive [11]. Eleven out of 20 grade II tumors showed PR positivity, and all grade III tumors were progesterone-negative tumors.

Twenty-eight out of 33 cases (84.8%) of stage I tumors were progesterone positive. PR positivity was seen in three out of six stage II tumors and two out of five stage III tumors. All stage IV cases were progesterone-negative tumors. In our study, 31 out of 59 cases (52.5%) showed combined ER and PR positivity which was 15% low compared to that reported in the literature which showed 68.9% of combined ER and PR positivity, and 12 out of 59 cases (20.33%) showed combined ER and PR negativity which was higher than the literature [11]. Most grade I and stage I tumors showed PR positivity. Both ER and PR are negative in high-grade, non-endometrioid, and advanced stages of endometrial carcinomas. There is an inverse correlation between estrogen receptors, progesterone receptors, and high-stage with high-grade tumors are noted. The progesterone receptor is a strong prognostic factor than the estrogen receptor.

In our study group, BCL-2 expression was seen in 38 out of 59 cases (64.4%) which is similar to the literature reports where 61.1% of cases were found to be BCL-2 positive. BCL-2 was positive in 30 out of 35 cases of (85.71%) grade I tumors which is similar to the literature [6]. A total of 40% of grade II tumors showed BCL-2 expression. All grade III tumors showed loss of expression of BCL-2.

Twenty-five out of 33 cases (75.75%) of stage I tumors, three out of six stages II tumors, and one out of five stages III tumors were positive for BCL-2. All stage IV tumors were BCL-2 negative which was similar to the literature [12]. Out of 33 ER-positive cases, 25 cases (55%) show BCL-2 expression which was higher than the ER-negative cases (45.9%). Also, significantly higher BCL-2 positivity was seen in PR-positive cases (34 out of 44) which accounts for 74% more than the PR-negative cases (26%). This was similar to the study done by Markova et al. [14]. In our study group, HER2/neu receptor was positive in six cases (10.1%) out of 59 cases. HER2/neu receptor was expressed only in two out of 55 (3.63%) cases of type I tumors. All four (100%) non-endometrioid adenocarcinomas expressed HER2/neu receptor which was similar to the literature, and HER2/neu receptor was positive in 10% (two out of 20) of grade II and 100% in grade III tumors, four out of five (80%) of stage III and all stage IV tumors were HER2/neu receptor-positive [11].

From our study, we inferred estrogen, progesterone receptor and BCL-2 were positive in grade I and stage I tumors. HER2-neu receptors were expressed in high-grade and higher-stage tumors. There was an inverse correlation between ER, PR, BCL-2, and HER2/neu receptors expression in endometrial carcinomas included in the study.

The limitation of our study includes a small sample size. Therefore a large prospective study on both curetting and hysterectomy specimens with long-term follow-up is essential to prove the utility of ER, PR, BCL-2, and HER2/neu expression as a routine prognostic tool.

## Conclusions

In conclusion, our study on endometrial carcinoma reported in the institute from January 2010 to December 2014 revealed 1.6% incidence of endometrial carcinoma among women. The majority of these occurred in the mean age group of 55 years and women below 40 years of age had type I endometrioid adenocarcinoma. In our study, we used immunohistochemical markers for ER, PR, BCL-2, and HER2/neu. There is no correlation between BCL-2 and ER, PR, HER2/neu expression seen in high-stage and high-grade tumors. BCL-2 expression is directly proportional to grade, stage, and hormone receptor status and inversely proportional to HER2/neu expression.

## References

[1] Srikantia N, Rekha B, Rajeev AG, Kalyan SN (2009). Endometrioid endometrial adenocarcinoma in a premenopausal woman with multiple organ metastases. Indian J Med Paediatr Oncol.

[2] Kim JW, Kim SH, Kim YT, Kim DK (2002). Clinicopathologic and biological parameters predicting the prognosis in endometrial cancer. Yonsei Med J.

[3] Bharatnur S, Kustagi P, Krishnamohan D (2011). Endometrial carcinoma in a young woman: "30 is not immune". J Obstet Gynaecol India.

[4] Tran AQ, Gehrig P (2017). Recent advances in endometrial cancer. F1000Res.

[5] Iqbal N, Iqbal N (2014). Human epidermal growth factor receptor 2 (HER2) in cancers: overexpression and therapeutic implications. Mol Biol Int.

[6] Sakuragi N, Ohkouchi T, Hareyama H (1998). BCL-2 expression and prognosis of patients with endometrial carcinoma. Int J Cancer.

[7] Mathur P, Sathishkumar K, Chaturvedi M (2020). Cancer statistics, 2020: report from National Cancer Registry Programme, India. JCO Glob Oncol.

[8] Balasubramaniam G, Sushama S, Rasika B, Mahantshetty U (2013). Hospital-based study of endometrial cancer survival in Mumbai, India. Asian Pac J Cancer Prev.

[9] Rodriguez AC, Blanchard Z, Maurer KA, Gertz J (2019). Estrogen signaling in endometrial cancer: a key oncogenic pathway with several open questions. Horm Cancer.

[10] Busmanis I, Ho TH, Tan SB, Khoo KS (2005). p53 and BCL-2 expression in invasive and pre-invasive uterine papillary serous carcinoma and atrophic endometrium. Ann Acad Med Singap.

[11] Maniketh I, Ravikumar G, Crasta JA, Prabhu R, Vallikad E (2014). Estrogen and progesterone receptor expression in endometrioid endometrial carcinomas: a clinicopathological study. Middle East J Cancer.

[12] Kalogiannidis I, Bobos M, Papanikolaou A (2008). Immunohistochemical BCL-2 expression, p53 overexpression, PR and ER status in endometrial carcinoma and survival outcomes. Eur J Gynaecol Oncol.

[13] Srijaipracharoen S, Tangjitgamol S, Tanvanich S (2010). Expression of ER, PR, and HER-2/neu in endometrial cancer: a clinicopathological study. Asian Pac J Cancer Prev.

[14] Markova I, Duskova M, Lubusky M, Kudela M, Zapletalová J, Procházka M, Pilka R (2010). Selected immunohistochemical prognostic factors in endometrial cancer. Int J Gynecol Cancer.

